# Comparison of the effect of hyaluronic acid injection versus extracorporeal shockwave therapy on chronic plantar fasciitis: Protocol for a randomized controlled trial

**DOI:** 10.1371/journal.pone.0250768

**Published:** 2021-06-24

**Authors:** Gabriel Ferraz Ferreira, Davy Sevilla, Carolinne Nascimento Oliveira, Luiz Carlos Nogueira Junior, Gustavo Gonçalves Arliani, Victor Otávio Oliveira, Miguel Viana Pereira Filho

**Affiliations:** 1 Foot and Ankle Surgery Group, Orthopaedics and Traumatology Unit, Prevent Senior, São Paulo, Brazil; 2 Department of Orthopaedics and Traumatology, Prevent Senior, São Paulo, Brazil; 3 Head of Department, Orthopaedics and Traumatology Unit, Prevent Senior, São Paulo, Brazil; 4 Head of Foot and Ankle Surgery Group, Orthopaedics and Traumatology Unit, Prevent Senior, São Paulo, Brazil; Prince Sattam Bin Abdulaziz University, College of Applied Medical Sciences, SAUDI ARABIA

## Abstract

**Background:**

Plantar fasciitis is the most common cause of pain in the plantar region of the heel, and extracorporeal shockwave therapy (ESWT) is an option used in cases where conservative treatment fails. Hyaluronic acid (HA), initially used for osteoarthrosis, is a treatment option because it has been applied to extra-articular regions, such as tendons, ligaments, and fascia. The aim of the present study will be to evaluate the outcomes of pain, function, and personal satisfaction after a single injection of HA and to compare the results with those of ESWT in patients with chronic plantar fasciitis.

**Methods:**

The study will include 80 patients who will be randomized to receive three sessions of ESWT (n = 40) or a single ultrasound-guided HA injection in the plantar fascia (n = 40). The outcomes will include the visual analog pain scale score, American Orthopaedic Foot and Ankle Society (AOFAS) score, and Foot and Ankle Outcome Score (FAOS). All of the assessments will be performed at baseline and 3, 6, and 12 months after treatment. Statistical analysis will be performed using the repeated measures ANOVA (analysis of variance test) for primary and secondary outcomes and also Fisher’s Least Significant Difference, a Post-Hoc test. We will use R software for statistical analysis, randomization, and sample size calculation.

**Results:**

Recruitment and data collection will begin in November 2020, with completion scheduled for November 2022 and final publication available in March 2023.

**Conclusion:**

This trial will evaluate the effects of a single ultrasound-guided HA injection for the treatment of chronic plantar fasciitis.

**Trial registration:**

Brazilian Clinical Trials Registry (Register Number: RBR-97vkx4) http://www.ensaiosclinicos.gov.br/rg/RBR-97vkx4/.

## Introduction

### Background

Plantar fasciitis is the most common cause of pain in the plantar region of the heel, especially in middle-aged and elderly patients [1], and is often described as an overload of the plantar fascia [2,3]. In most cases, plantar fasciitis is self-limiting, but the time for full resolution of symptoms can take up to one year, impairing the quality of life of patients and leading to frustration among the healthcare team [4].

Several treatments have been described for plantar fasciitis, and the most commonly used are noninvasive, such as nonsteroidal anti-inflammatory drugs, analgesics, night orthoses, stretching, exercises, and insoles [5].

In cases of conservative treatment failure, some studies have shown benefits of corticosteroid injection, resulting in rapid improvement and ease of progression during rehabilitation [6,7]. However, the use of corticosteroids can cause problems such as rupture of the plantar fascia, infection, atrophy of the heel pad, and even changes in skin pigmentation [8–10].

Thus, extracorporeal shockwave therapy (ESWT) has emerged as an option for recalcitrant cases of plantar fasciitis without improvement with conventional conservative treatment. This modality showed satisfactory results in a placebo-controlled study [11] but was dependent on the intensity, pulse cycle, and shockwave modality [12].

Meanwhile, due to successful treatment of knee arthrosis through hyaluronic acid (HA) injection [13], the possibility of expanding its indication for regions such as the fascia and tendons has arisen, and chronic plantar fasciitis is a possible candidate for this treatment due to its anti-inflammatory and healing potential [14,15].

### Study aim and hypotheses

The objective of the study is to compare the therapeutic efficacy of ultrasound-guided HA application for chronic plantar fasciitis with that of ESWT. We suggest that recalcitrant plantar fasciitis may benefit from the anti-inflammatory and healing properties of HA, thus accelerating the process of symptom improvement. The comparison will be with the ESWT because there are several studies proving its benefit, representing an excellent control group.

## Methods

### Study design

**Fig 1:**
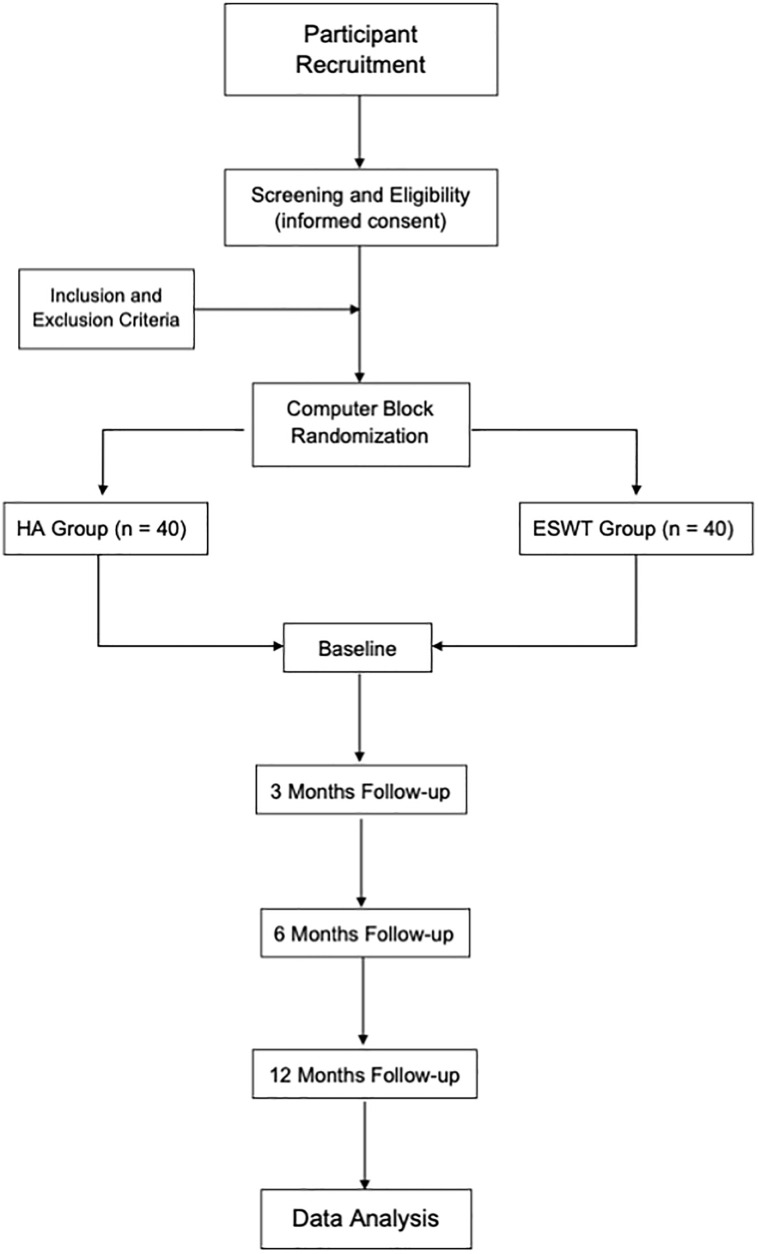
Research flow diagram.

This study is designed as a randomized, controlled, parallel groups, and intervention trial (Fig 1).

### Recruitment

The participants will be recruited during routine orthopedic outpatient care provided by the authors at the Anália Franco and Bento Pires Unit of Prevent Senior, São Paulo, Brazil. Recruitment will be continuous and sequenced in order to select a cohort of more heterogeneous patients in the study.

### Ethics

This protocol was approved by Scientific Research Committee of Instituto Prevent Senior (Number 3,912,441–2020) and is registered on the Brazilian Clinical Trials Registry [16] (Register Number: RBR-97vkx4). The authorization to use and disclose participant information is restricted to this study, and the data will be kept for five years after publication.

### Eligibility

The inclusion criteria are as follows: 1) clinical diagnosis of plantar fasciitis with failure of conservative treatment including stretching exercises, nonsteroidal anti-inflammatory drugs, analgesics, and insoles for at least three months; 2) individuals of both genders who are older than 18 years and younger than 80 years; 3) the patient must be able to understand the informed consent form; 4) visual analog pain scale (VAS) score greater than or equal to three points (range from 0 to 10 points).

The exclusion criteria are patients with neuropathy, skin lesions, previous degenerative lesions of the ankle, previous surgeries of the ankle or Achilles tendon, previous allergy to sodium hyaluronate, or allergies to avian proteins, those less than 16 years of age, and patients with sequelae of fractures of the tibial pilon, ankle, or foot. In addition, patients with collagen disorders, rheumatoid arthritis, and seronegative arthritis will be excluded. Patients who are not recommended to use shockwave therapy include the following: patients who use anticoagulants, are pregnant, have a tumor in the treatment area, systemic infections or epilepsy.

### Handling of data

The data management plan aims to synthesize high-quality data through standardized procedures and thus, reduce errors and missing data that occur, generating an ideal database for analysis.

Patient data will be collected in order to use numbers for personal identification (ID) and managed using REDCap electronic data capture tools hosted at Instituto Prevent Senior [17,18]. Full access to the final test data of the study will be granted only to selected researchers.

REDCap (Research Electronic Data Capture) is a secure, web-based software platform designed to support data capture for research studies, providing 1) an intuitive interface for validated data capture; 2) audit trails for tracking data manipulation and export procedures; 3) automated export procedures for seamless data downloads to common statistical packages; and 4) procedures for data integration and interoperability with external sources. All data will be available to the public in a data repository, without personal identification of the participants, after publication of the study.

### Missing data

We consider minimal or no follow-up losses for the primary outcome. If more than 5% of the missing data in the primary outcome is identified, we will perform a sensitivity analysis using multiple imputations and estimation equation methods.

### Sample size

The sample size was calculated using the *pwr* package of R software [19]. A study by Chew et al [20] was used to determine the minimum change required in the treatment of chronic plantar fasciitis with regard to the main outcome, which will consist of a change of 3.0 points in the VAS between the baseline value and that at three months of follow-up after the intervention. The pooled standard deviation was set to 4.5 after examining preliminary values. Thus, we chose a study power of 80% and type I error of 5%. The calculated sample size was 36 patients in each group, but a sample size of 40 was specified to allow loss to follow-up during the study (10%).

### Randomization

Participants (n = 80) who meet all eligibility criteria will be randomized at a 1:1 ratio to either the HA intervention group or the control group (ESWT). Randomization will be performed in blocks (random sizes of two, four, six, and eight) and used to ensure that the treatment groups is balanced throughout the study period. This strategy ensures that the intervention group and control group are balanced regarding the number of participants. To ensure allocation concealment, random variation in block sizes (four to eight participants per block) will be used. We will use the *blockrand* package of the R software to generate the randomization table.

### Intervention

The interventions will be performed in the authors’ outpatient clinic and only after the participants sign the informed consent form and are allocated to one of the study arms.

The intervention with HA will be performed by the author (G.F.F.) through a single application of Ostenil Plus^®^ (2.0 mL with 40 mg of 2.0% sodium hyaluronate, TRB Chemedica, Munich, Germany). This product is indicated for extra-articular regions, as indicated in the package insert: “It is also indicated in cases of pain and mobility restriction due to inflammation and degeneration in tendons, tendon sheaths, and entheses.” This product contains low-molecular-weight HA. The injection will be guided in all cases by a portable ultrasound Butterfly iQ^®^ device (Butterfly Network, New York, USA) to increase the precision and efficacy of the intervention.

The ESWT intervention (radial type) will be performed by the author (L.C.N.J.) in three sessions in total, one per week, using a Swiss DolorClast Master^®^ device (Electro Medical System, Nyon, Switzerland). Patients will be subjected to 2,500 focused shockwaves with an energy flux density of 0.05 mJ/mm^2^ and a frequency of 8 Hz. Each session will last approximately 15 min. The treatment will follow that recommended by the generator’s distribution company.

After the interventions, participants will be recommended to perform daily posterior chain stretching exercises while remaining under follow-up with the physical therapy team. The use of oral analgesics will be prescribed and authorized.

Regarding the risks of the interventions, local injection of HA may cause adverse effects such as pain, sensation of heat, redness, and edema, as described in the package insert. Shockwave therapy may cause an inflammatory process and local pain, but these symptoms are usually quickly resolved with mild analgesics.

### Measures

Clinical evaluation using pain, function, and personal satisfaction scores will be performed for a period of one year in the following intervals after the interventions: three, six, and 12 months. Thus, we will be able to evaluate the results in the short, medium, and long term. The authors (X.X.X.X., X.X.X.) will blindly evaluate the participants regarding the intervention performed.

### Outcome measures

The primary outcome to be evaluated will be the VAS. The questionnaire will be administered at baseline and three, six, and 12 months after the intervention during routine visits previously scheduled with the researchers.

The secondary outcomes to be evaluated will be function and personal satisfaction. The former will be assessed based on the American Orthopaedic Foot and Ankle Society (AOFAS) score [21] for the ankle and hindfoot and the Foot and Ankle Outcome Score (FAOS) [22]. The latter will be evaluated based on the personal satisfaction criteria published by Roles and Maudsley [23].

### Statistical analysis

Statistical analysis will be conducted primarily through an intention-to-treat analysis of all patients, in both arms, except for lost cases or withdrawal of informed consent. In addition, a per–protocol analysis will be conducted.

All analysis will be performed using the statistics package of R software [19]. We will use the repeated measures ANOVA (analysis of variance test) for the primary and secondary outcomes and also the Fisher’s Least Significant Difference, a Post-Hoc test.

Continuous variables will be measured using descriptive statistics, including the mean and standard deviation, and tested for the normality of their distribution using the Shapiro test [24]. Categorical variables will be presented according to their proportion and confidence interval.

## Results

Recruitment and data collection started in November 2020. We estimate that data collection should be completed in June 2022, and the results should be available in November 2022.

## Discussion

### Overview

The present study will be unprecedented in terms of the inclusion of HA injection for the treatment of chronic plantar fasciitis in a randomized clinical trial. We believe that use of shockwave therapy in the control group can serve as a reliable parameter in comparing the results.

The possibility of comparing the results with those of corticosteroid injection seems to be the most rational, but the published results on complications related to rupture and injury of the plantar fascia contributed to the decision to not include this type of intervention in the study design [8–10].

We also chose not to include placebo therapies such as anesthetic infiltration or needling because shockwave therapy has shown satisfactory results and thus can be used as a control. The use of HA in the study group is justified because it is a new procedure for treatment of chronic plantar fasciitis, with only one case series showing benefits with its use [14].

In addition, HA has been increasingly used in the treatment of tendinopathies and extra-articular inflammation [25–27]. Other types of interventions, such as botulinum toxin injection, were excluded based on previously published results [28].

The described effects of HA include local lubrication [29], promoting tissue healing in the region between bone and tendon [30], and tissue regeneration [31]. Another important function reported is to decrease the sensitivity of regional sensory nerves, with decreasing pain [32].

The ESWT intervention produce an effect that is considered direct and other indirect on the treated region. The indirect effect is the production of localized cavity bubbles. The direct effect is the transmission of energy from the equipment to the target tissue. Both produce a local biological response [33].

This protocol describes a study focused on the application of a new treatment method to combat chronic plantar fasciitis. The results of this study will have important implications for the prescription of HA as a therapeutic option, showing whether clinical improvement occurs and comparing the results with those of an effective treatment method.

### Strengths and limitations of this study

The strengths of the study are as follows: 1) new treatment method; 2) appropriate methodological design; 3) previous publication of the protocol, thus minimizing publication bias; and 4) single-center study using the same technique, interventions, and evaluators. We believe that the main limitations of the study are the short follow-up duration and that the mean age of patients will be greater than that of the general population (our center specializes in elderly patients).

### Conclusions

This trial will evaluate the effects of a single ultrasound-guided hyaluronic acid injection for the treatment of chronic plantar fasciitis. The control group will receive an intervention using shockwave therapy, which has been shown to be effective in several previously published studies.

## Supporting information

S1 Checklist (DOC)

S1 File Ethics committee portuguese. (PDF)

S2 File Ethics committee english. (PDF)

S3 File Correction ethics committee portuguese. (PDF)

S4 File Correction ethics committee english. (PDF)

S5 File Complete project approved in portuguese. (PDF)

S6 File Complete project approved in english. (PDF)

## References

[1] Thomson CE, Crawford F, Murray GD. The effectiveness of extra corporeal shock wave therapy for plantar heel pain: a systematic review and meta-analysis. BMC Musculoskelet Disord. 2005;6:19. Epub 2005/04/26. doi: 10.1186/1471-2474-6-19 . 15847689 PMC1097736

[2] Kibler WB, Goldberg C, Chandler TJ. Functional biomechanical deficits in running athletes with plantar fasciitis. Am J Sports Med. 1991;19(1):66–71. Epub 1991/01/01. doi: 10.1177/036354659101900111 . 1672577

[3] Rompe JD. Plantar fasciopathy. Sports medicine and arthroscopy review. 2009;17(2):100–4. doi: 10.1097/JSA.0b013e3181a3d60e 19440137

[4] Buchbinder R. Plantar Fasciitis. New England Journal of Medicine. 2004;350(21):2159–66. doi: 10.1056/NEJMcp032745 . 15152061

[5] Puttaswamaiah R, Chandran P. Degenerative plantar fasciitis: A review of current concepts. Foot. 2007;17(1):3–9. doi: 10.1016/j.foot.2006.07.005

[6] Genc H, Saracoglu M, Nacir B, Erdem HR, Kacar M. Long-term ultrasonographic follow-up of plantar fasciitis patients treated with steroid injection. Joint Bone Spine. 2005;72(1):61–5. Epub 2005/02/01. doi: 10.1016/j.jbspin.2004.03.006 . 15681250

[7] Kiter E, Celikbas E, Akkaya S, Demirkan F, Kilic BA. Comparison of injection modalities in the treatment of plantar heel pain: a randomized controlled trial. J Am Podiatr Med Assoc. 2006;96(4):293–6. Epub 2006/07/27. doi: 10.7547/0960293 . 16868321

[8] Acevedo JI, Beskin JL. Complications of plantar fascia rupture associated with corticosteroid injection. Foot Ankle Int. 1998;19(2):91–7. Epub 1998/03/14. doi: 10.1177/107110079801900207 . 9498581

[9] Sellman JR. Plantar fascia rupture associated with corticosteroid injection. Foot Ankle Int. 1994;15(7):376–81. Epub 1994/07/01. doi: 10.1177/107110079401500706 . 7951973

[10] Speed CA. Injection therapies for soft-tissue lesions. Best Pract Res Clin Rheumatol. 2007;21(2):333–47. Epub 2007/05/22. doi: 10.1016/j.berh.2006.11.001 . 17512486

[11] Ogden JA, Alvarez RG, Levitt RL, Johnson JE, Marlow ME. Electrohydraulic high-energy shock-wave treatment for chronic plantar fasciitis. J Bone Joint Surg Am. 2004;86(10):2216–28. Epub 2004/10/07. doi: 10.2106/00004623-200410000-00013 . 15466731

[12] Gerdesmeyer L, Frey C, Vester J, Maier M, Weil L Jr, Weil L Sr, et al. Radial extracorporeal shock wave therapy is safe and effective in the treatment of chronic recalcitrant plantar fasciitis: results of a confirmatory randomized placebo-controlled multicenter study. Am J Sports Med. 2008;36(11):2100–9. Epub 2008/10/04. doi: 10.1177/0363546508324176 . 18832341

[13] Altman R, Lim S, Steen RG, Dasa V. Hyaluronic Acid Injections Are Associated with Delay of Total Knee Replacement Surgery in Patients with Knee Osteoarthritis: Evidence from a Large U.S. Health Claims Database. PLoS One. 2015;10(12):e0145776. Epub 2015/12/24. doi: 10.1371/journal.pone.0145776 . 26694145 PMC4687851

[14] Kumai T, Samoto N, Hasegawa A, Noguchi H, Shiranita A, Shiraishi M, et al. Short-term efficacy and safety of hyaluronic acid injection for plantar fasciopathy. Knee Surg Sports Traumatol Arthrosc. 2018;26(3):903–11. Epub 2017/03/04. doi: 10.1007/s00167-017-4467-0 . 28255655

[15] Petrella RJ, Petrella MJ, Cogliano A. Periarticular hyaluronic acid in acute ankle sprain. Clin J Sport Med. 2007;17(4):251–7. Epub 2007/07/11. doi: 10.1097/JSM.0b013e3180f6169f . 17620777

[16] Saúde Md. Brazilian Clinical Trials Registry Brazil2020. http://www.ensaiosclinicos.gov.br.

[17] Harris PA, Taylor R, Thielke R, Payne J, Gonzalez N, Conde JG. Research electronic data capture (REDCap)—A metadata-driven methodology and workflow process for providing translational research informatics support. Journal of Biomedical Informatics. 2009;42(2):377–81. doi: 10.1016/j.jbi.2008.08.010 18929686 PMC2700030

[18] Harris PA, Taylor R, Minor BL, Elliott V, Fernandez M, O’Neal L, et al. The REDCap consortium: Building an international community of software platform partners. Journal of Biomedical Informatics. 2019;95:103208. doi: 10.1016/j.jbi.2019.103208 31078660 PMC7254481

[19] Team RC. R: A language and environment for statistical computing. Vienna, Austria: R Foundation for Statistical; 2014.

[20] Chew KT, Leong D, Lin CY, Lim KK, Tan B. Comparison of autologous conditioned plasma injection, extracorporeal shockwave therapy, and conventional treatment for plantar fasciitis: a randomized trial. PM R. 2013;5(12):1035–43. Epub 2013/08/27. doi: 10.1016/j.pmrj.2013.08.590 . 23973504

[21] Kitaoka HB, Alexander IJ, Adelaar RS, Nunley JA, Myerson MS, Sanders M. Clinical rating systems for the ankle-hindfoot, midfoot, hallux, and lesser toes. Foot Ankle Int. 1994;15(7):349–53. Epub 1994/07/01. doi: 10.1177/107110079401500701 . 7951968

[22] Imoto AM, Peccin MS, Rodrigues R, Mizusaki JM. Tradução e validação do questionário FAOS—FOOT and ankle outcome score para língua portuguesa. Acta Ortopédica Brasileira. 2009;17:232–5.

[23] Roles NC, Maudsley RH. Radial tunnel syndrome: resistant tennis elbow as a nerve entrapment. J Bone Joint Surg Br. 1972;54(3):499–508. Epub 1972/08/01. . 4340924

[24] SHAPIRO SS, WILK MB. An analysis of variance test for normality. Biometrika. 1965;52(3–4):591–611.

[25] Gallorini M, Berardi AC, Berardocco M, Gissi C, Maffulli N, Cataldi A, et al. Hyaluronic acid increases tendon derived cell viability and proliferation in vitro: comparative study of two different hyaluronic acid preparations by molecular weight. Muscles Ligaments Tendons J. 2017;7(2):208–14. Epub 2017/12/22. doi: 10.11138/mltj/2017.7.2.208 . 29264330 PMC5725168

[26] Honda H, Gotoh M, Kanazawa T, Ohzono H, Nakamura H, Ohta K, et al. Hyaluronic Acid Accelerates Tendon-to-Bone Healing After Rotator Cuff Repair. Am J Sports Med. 2017;45(14):3322–30. Epub 2017/09/06. doi: 10.1177/0363546517720199 . 28872895

[27] Kaux JF, Samson A, Crielaard JM. Hyaluronic acid and tendon lesions. Muscles Ligaments Tendons J. 2015;5(4):264–9. Epub 2016/03/10. . 26958533 10.11138/mltj/2015.5.4.264 PMC4762636

[28] Placzek R, Deuretzbacher G, Meiss AL. Treatment of chronic plantar fasciitis with Botulinum toxin A: preliminary clinical results. Clin J Pain. 2006;22(2):190–2. Epub 2006/01/24. doi: 10.1097/01.ajp.0000169674.34191.0e . 16428954

[29] Greenberg DD, Stoker A, Kane S, Cockrell M, Cook JL. Biochemical effects of two different hyaluronic acid products in a co-culture model of osteoarthritis. Osteoarthritis Cartilage. 2006;14(8):814–22. Epub 2006/04/18. doi: 10.1016/j.joca.2006.02.006 . 16617026

[30] Yagishita K, Sekiya I, Sakaguchi Y, Shinomiya K, Muneta T. The effect of hyaluronan on tendon healing in rabbits. Arthroscopy. 2005;21(11):1330–6. Epub 2005/12/06. doi: 10.1016/j.arthro.2005.08.020 . 16325083

[31] Mrosek E, Erggelet C, McDonald JA, Kurz H. Hyaluronan synthases in normal and regenerating joint cartilage. Cells Tissues Organs. 2003;173(2):93–104. Epub 2003/03/22. doi: 10.1159/000068944 . 12649587

[32] Gomis A, Miralles A, Schmidt RF, Belmonte C. Nociceptive nerve activity in an experimental model of knee joint osteoarthritis of the guinea pig: effect of intra-articular hyaluronan application. Pain. 2007;130(1–2):126–36. Epub 2007/01/02. doi: 10.1016/j.pain.2006.11.012 . 17197090

[33] Chinnery J, Faust A, Siebert W, Buch M. Extracorporeal shock waves in orthopaedics: Springer Science & Business Media; 2012.

