# Supplementary material for: Comparison of the effect of hyaluronic acid injection versus extracorporeal shockwave therapy on chronic plantar fasciitis: Protocol for a randomized controlled trial
Source: PLoS One. 2021 Jun 24;16(6):e0250768. doi: 10.1371/journal.pone.0250768 (PMC8224905; doi:10.1371/journal.pone.0250768)
Supplement: S1 File — (PDF) [file pone.0250768.s002.pdf]

Research project:
Comparison of the effect of hyaluronic acid injection versus extracorporeal shock wave therapy in chronic plantar fasciitis: a randomized clinical trial

## Preliminary Information

### Principal Responsible

| | |
|---|---|
| CPF/Documento: 354.474.218-70 | Name: GABRIEL FERRAZ FERREIRA |
| Telefone: 1135547084 | E-mail: gabriel.ferraz38@yahoo.com.br |

### Proponent Institution

| | |
|---|---|
| CNPJ: 00.461.479/0031-89 | Institution Name: PREVENT SENIOR PRIVATE OPERADORA DE SAUDE LTDA |

**Is it an international study?** No

### Research Team

| CPF/Documento | Nome |
|---|---|
| 268.802.128-16 | Miguel Viana Pereira Filho |

## Study area

**Grandes Áreas do Conhecimento (CNPq)**

- Grande Área 4. Ciências da Saúde

**Propósito Principal do Estudo (OMS)**

- Clínico

**Public Search Title:** Comparison of the effect of hyaluronic acid injection versus extracorporeal shock wave therapy in chronic plantar fasciitis: a randomized clinical trial

### Contato Público

| CPF/Documento | Nome | Telefone | E-mail |
|---|---|---|---|
| 354.474.218-70 | GABRIEL FERRAZ FERREIRA | 1135547084 | gabriel.ferraz38@yahoo.com.br |

**Contato Científico:** GABRIEL FERRAZ FERREIRA

## Desenho de Estudo / Apoio Financeiro

Desenho do Estudo: Intervenção/Experimental

### Health conditions or problems

| Condição de saúde ou Problema |
|---|
| Plantar Fasciitis |
| Hyaluronic acid |
| Shockwaves |

### General Descriptors for Health Conditions

ICD1-10: International Classification of Diseases

| Código CID | Descrição CID |
|---|---|
| M25.5 | Dor articular |

DeCS:Descritores em Ciência da Saúde

| Código DECS | Descrição DECS |
|---|---|
| 32350 | Artralgia |

### Descritores Específicos para as Condições de Saúde

CID1-10:Classificação Internacional de Doenças

| Código CID | Descrição CID |
|---|---|
| M77.3 | Esporao do calcaneo |

DeCS:Descritores em Ciência da Saúde

| Código DECS | Descrição DECS |
|---|---|
| 37270 | Fascite Plantar |

Tipo de Intervenção: Comparador

**Natureza da Intervenção**

- Fármaco/Medicamento/Vacina

### Intervention Descriptors

Descritores da Intervenção

| Intervenções |
|---|
| Intervention Descriptors |

Lista de CID

| Código CID | Descrição CID |
|---|---|
| M77.3 | Esporao do calcaneo |

Lista de DECS

| Código DECS | Descrição DECS |
|---|---|
| 37270 | Fascite Plantar |
| 57173 | Tratamento por Ondas de Choque Extracorpóreas |

**Fase**

- Fase 1

Design:
The present study will be a prospective randomized controlled clinical trial, whose treatment effectiveness will be evaluated by comparing two different groups: treatment group that will receive sodium hyaluronate and the shock wave treatment group. The patients included in the study will come from the outpatient clinic of the Orthopedics and Traumatology Unit of Hospital Sancta Maggiore Mooca. The objective of the study is to compare pain outcomes: visual analog scale (VAS) 1, mobility function (AOFAS) 2, and to determine the effectiveness of these treatments (Appendix 1, 2).

### Apoio Financeiro

| CNPJ | Nome | E-mail | Telefone | Tipo |
|---|---|---|---|---|
| | | | | Financiamento |

## Keywords

| Keywords |
|---|
| Plantar Fasciitis |
| Hyaluronic acid |
| Extracorporeal Shock Wave Treatment |

## Study Detail

Resume:
Plantar fasciitis is an extremely common condition in orthopedists' daily lives. There are numerous non-invasive treatments described, but in some cases they do not show any effect and can evolve to the chronicity of the lesion. Thus, some more invasive methods such as infiltration of the plantar fascia with hyaluronic acid. Hyaluronic acid is widely used in arthrosis of the knee and other joints as an option to delay surgical treatment and as an adjunct to conservative treatment. The biochemical properties of this substance guarantee a decrease in the inflammatory process as well as nourishing the articular cartilage. Thus, the infiltration of sodium hyaluronate can act as an inflammatory and analgesic mediator, avoiding the common complications caused by infiltration with corticosteroids. Another option for the treatment of chronic plantar fasciitis is shockwave therapy, with several studies demonstrating its effectiveness. The aim of this study is to compare the therapeutic effect of the single application of sodium hyaluronate for chronic plantar fasciitis guided by ultrasound with shock wave therapy.

Introduction:

The use of sodium hyaluronate is an important non-surgical treatment for knee arthrosis, being an option in conservative treatment. This substance has characteristics as a mediator of inflammation, reducing the passage of these cells to the applied site.3-5

The use of hyaluronic acid has also been described for the treatment of pathologies related to extra-articular inflammations, such as tendons, ligaments and fascias. Excessive stress can be concentrated at the interface between structures with different mechanical properties, causing a variety of pathological conditions.6

The most used treatment for enthesopathies and specifically plantar fasciitis include posterior chain stretching, physiotherapy and the use of non-steroidal anti-inflammatory drugs.5-7 Another option for the treatment of chronic plantar fasciitis is shock wave therapy, with several studies demonstrating its effectiveness.8-11

The aim of the study is to compare the therapeutic effectiveness of applying sodium hyaluronate for chronic plantar fasciitis guided by ultrasound with shock wave therapy.

Hypothesis:
It is believed that sodium hyaluronate may allow for better physical rehabilitation with less pain and greater mobility, avoiding costly surgical procedures and possible risks of complications.

Primary Objective:
The primary objective of the study is to evaluate the analgesic and anti-inflammatory effect of the single application of sodium hyaluronate for chronic plantar fasciitis and to compare it with shock wave therapy.

Secondary Objective:
The secondary objective of the study is to assess foot function and satisfaction criteria after the single infiltration of sodium hyaluronate for chronic plantar fasciitis and to compare it with shock wave therapy.

Proposed Methodology:

The study will include patients diagnosed with plantar fasciitis in the outpatient clinics of the orthopedics and traumatology unit of Hospital Sancta Maggiore Mooca who did not improve their symptoms with at least three months of conservative treatment.

Patients will be invited to participate in the study, and the informed consent form (ICF) will be presented. Only individuals who sign the term and agree with the evaluation methods will be included.

After that, such patients will be randomized to the infiltration group with hyaluronic acid or to the shock wave therapy group. The treatments will be carried out in the outpatient clinic of the hospital. Hyiluronic acid will be applied by the same health professional. Treatment with shock waves, on the other hand, will be carried out by another health professional. Clinical evaluation with application of pain, function and personal satisfaction scores will be carried out for 24 months at the following consultation intervals: 1, 3, 6, 12 and 24.

The sample size was calculated using the “pwr” package of the R software with a prediction for the “T test” according to the parameters: 0.75 power test, 0.3 effect size (proposed by Cohen) and level of significance of 0.05, with 80 participants considered necessary.

In this way, we will be able to evaluate the result in the short, medium and long term. Associated with therapies, patients will remain in rehabilitation work with physiotherapy. The data will be documented in the Excel program, and then the data will be analyzed. The work will be submitted to the local Ethics Committee according to the guidance of Plataforma Brasil.

There will be no cost for the health operator or for the patient himself in this evaluation since it will be carried out in routine appointments previously scheduled in the outpatient clinic of the author and co-authors.

Randomization will be determined using a randomized block table generated by comp

but is usually quickly resolved with mild pain relievers.
Benefits:
The infiltration of the plantar fascia with sodium hyaluronate presents advantages described as in pain control, replacement of peritendinous fluid, which can provide prolonged comfort and better rehabilitation. Shock wave therapy has an excellent benefit for plantar fasciitis as described in the literature.
Data Analysis Methodology:
The associations will be tested by the Mann-Whitney comparative test of means with a 5% significance level, after evaluating the distribution of the samples by the Kolmogorov-Smirnov test, and therefore, those whose p value was less than 0.05. We will use Chi-square or Fischer's exact test to assess categorical variables. Statistical analysis will be performed using the stats package for software R.
Primary Outcome:
This technique can be used in chronic plantar fasciitis as an adjunct to analgesia and better rehabilitation, avoiding invasive and costly surgical procedures.

**Tamanho da Amostra no Brasil:** 80

**Data do Primeiro Recrutamento:** 02/03/2021

**Recruitment Countries**

| País de Origem do Estudo | País | Nº de participantes da pesquisa |
|---|---|---|
| Sim | BRASIL | 80 |

**Other information**

**Will secondary data sources (medical records, demographic data, etc.) be used?**
**No**
**Inform the number of individuals approached personally, recruited, or who will undergo any type of intervention in this research center:**
**80**
**Groups into which the research participants at this center will be divided**

| ID Grup | No. of Individuals | Interventions to be carried out |
|---|---|---|
| Hyaluronic acid | 40 | Infiltration of the plantar fascia guided by ultrasound with hyaluronic acid |
| Extracorporeal Shock Wave Treatment | 40 | Three sessions of extracorporeal shock wave therapy |

**Is the Study Multicentric in Brazil?**

**No**

**Do you propose exemption from IC?**

**No**

**Will there be retention of samples for bank storage?**

**Execution cronogram**

| Identificação d Etapa | Início (DD/MM/AAAA) | Término (DD/MM/AAAA) |
|---|---|---|
| Envio do Projeto a Comissão de ética | 01/11/2019 | 01/12/2019 |
| Elaboração do projeto | 23/10/2019 | 30/10/2019 |
| Análise dos resultados e correção estatística | 01/02/2021 | 28/02/2021 |
| Idealização do tema | 23/10/2019 | 30/10/2019 |
| Elaboração do artigo para publicação | 01/03/2021 | 01/05/2021 |
| Envio do artigo para publicação | 01/06/2021 | 01/07/2021 |
| Coleta de dados | 01/03/2021 | 01/01/2022 |

**Orçamento Financeiro**

| Identificação de Orçamento | Tipo | Valor em Reais (R$) |
|---|---|---|
| Papelaria | Custeio | R$ 200,00 |
| Total em R$ | | R$ 200,00 |

**References:**

1. Downie W, Leatham P, Rhind V, Wright V, Branco J, Anderson J. Studies with pain rating scales. Ann Rheum Dis 1978;37(4):378-81. 2. Coughlin MJ. Treatment of bunionette deformity with longitudinal diaphyseal osteotomy with distal soft tissue repair. Foot Ankle 1991;11(4):195-203. 3. Kitaoka HB, Alexander IJ, Adelaar RS, Nunley JA, Myerson MS, Sanders M. Clinical rating systems for the ankle-hindfoot, midfoot, hallux, and lesser toes. Foot Ankle Int 1994;15(7):349-53. doi: 10.1177/107110079401500701. 4. Altman R, Lim S, Steen RG, Dasa V. Hyaluronic acid injections are associated with delay of total knee replacement surgery in patients with knee osteoarthritis: evidence from a large U.S. health claims database. PLoS One 10:e0145776, 2015. 5. Saw KY, Hussin P, Loke SC, Azam M, Chen HC, Tay YG, Low S, Wallin KL, Ragavanaidu K. Articular cartilage regeneration with autologous marrow aspirate and hyaluronic acid: an experimental study in a goat model. Arthroscopy 25:1391–1400, 2009. 6. Petrella RJ, Petrella MJ, Cogliano A. Periarticular hyaluronic acid in acute ankle sprain. Clin J Sport Med 17:251–257, 2007. 7. Petrella MJ, Cogliano A, Petrella RJ. Original research: long-term efficacy and safety of periarticular hyaluronic acid in acute ankle sprain. Phys Sportsmed 37:64–70,2009. 8. Böddeker R, Schäfer H, Haake M (2001) Extracorporeal shockwave therapy (ESWT) in the treatment of plantar fasciitis–a biometrical review. Clin Rheumatol 20(5):324–330 9. Buchbinder R, Ptasznik R, Gordon J et al (2002) Ultrasoundguided extracorporeal shock wave therapy for plantar fasciitis: a randomized controlled trial. JAMA 288(11):1364–1372 10. Basford JR, Malanga GA, Krause DA et al (1998) A randomized controlled evaluation of low-intensity laser therapy: plantar fasciitis. Arch Phys Med Rehabil 79(3):249–254 11. Kamel M, Kotob H (2000) High frequency ultrasonographic findings in plantar fasciitis and assessment of local steroid injection. J Rheumatol 27(9):2139–2141

## Upload de Documentos

**Arquivo Anexos:**

| Tipo | Arquivo |
|---|---|
| Projeto Detalhado / Brochura Investigador | PROJETO_PESQUISA.pdf |
| Declaração de Pesquisadores | Declaracao_do_Pesquisador.pdf |
| Cronograma | CRONOGRAMA.pdf |
| Outros | Parecer.pdf |
| Folha de Rosto | FolhaDeRosto.pdf |
| TCLE / Termos de Assentimento / Justificativa de Ausência | TCLE.pdf |
| Outros | APRESENTACAO.pdf |
| Declaração de Instituição e Infraestrutura | infraestrutura.pdf |
| Orçamento | ORCAMENTO.pdf |

## Finalizar

Manter sigilo da integra do projeto de pesquisa: Não
