# Supplementary material for: Comparison of the effect of hyaluronic acid injection versus extracorporeal shockwave therapy on chronic plantar fasciitis: Protocol for a randomized controlled trial
Source: PLoS One. 2021 Jun 24;16(6):e0250768. doi: 10.1371/journal.pone.0250768 (PMC8224905; doi:10.1371/journal.pone.0250768)
Supplement: S2 File — (PDF) [file pone.0250768.s003.pdf]

Projeto de Pesquisa:
A comparação do efeito da injeção de ácido hialurônico versus terapia por ondas de choque extracorpórea na fascite plantar crônica: ensaio clínico randomizado

Informações Preliminares

Responsável Principal

CPF/Documento: 354.474.218-70	Nome: GABRIEL FERRAZ FERREIRA
Telefone: 1135547084	E-mail: gabriel.ferraz38@yahoo.com.br

Instituição Proponente

CNPJ: 00.461.479/0031-89	Nome da Instituição: PREVENT SENIOR PRIVATE OPERADORA DE SAUDE LTDA

É um estudo internacional? Não

Equipe de Pesquisa

CPF/Documento	Nome
268.802.128-16	Miguel Viana Pereira Filho

Área de Estudo

Grandes Áreas do Conhecimento (CNPq)

- Grande Área 4. Ciências da Saúde

Propósito Principal do Estudo (OMS)

- Clínico

Título Público da Pesquisa: A comparação do efeito da injeção de ácido hialurônico versus terapia por ondas de choque extracorpórea na fascite plantar crônica: ensaio clínico randomizado

Contato Público

CPF/Documento	Nome	Telefone	E-mail
354.474.218-70	GABRIEL FERRAZ FERREIRA	1135547084	gabriel.ferraz38@yahoo.com.br

Contato Científico: GABRIEL FERRAZ FERREIRA

Desenho de Estudo / Apoio Financeiro

Desenho do Estudo: Intervenção/Experimental

Condições de saúde ou problemas

Condição de saúde ou Problema
Fascite Plantar
Ácido Hialurônico
Ondas de Choque

Descritores Gerais para as Condições de Saúde

CID1-10:Classificação Internacional de Doenças

Código CID	Descrição CID
M25.5	Dor articular

DeCS:Descritores em Ciência da Saúde

Código DECS	Descrição DECS
32350	Artralgia

Descritores Específicos para as Condições de Saúde

CID1-10:Classificação Internacional de Doenças

Código CID	Descrição CID
M77.3	Esporao do calcaneo

DeCS:Descritores em Ciência da Saúde

Código DECS	Descrição DECS
37270	Fascite Plantar

Tipo de Intervenção: Comparador

Natureza da Intervenção

- Fármaco/Medicamento/Vacina

Descritores da Intervenção

Descritores da Intervenção

Intervenções
Ácido Hialurônico

Lista de CID

Código CID	Descrição CID
M77.3	Esporao do calcaneo

Lista de DECS

Código DECS	Descrição DECS
37270	Fascite Plantar
57173	Tratamento por Ondas de Choque Extracorpóreas

Fase

- Fase 1

Desenho:

O presente estudo será um ensaio clínico randomizado controlado, prospectivo, cuja eficácia do tratamento será avaliada por comparação entre dois grupos distintos: grupo de tratamento que receberá hialuronato de sódio e o grupo de tratamento por ondas de choque. Os pacientes incluídos no estudo serão provenientes do ambulatório da Unidade de ortopedia e traumatologia do Hospital Sancta Maggiore Mooca. O Objetivo do estudo é comparar os desfechos de dor: escala visual analógica (EVA)1, função mobilidade (AOFAS)2, e determinar a eficácia desses tratamentos (Apêndice 1, 2).

Apoio Financeiro

CNPJ	Nome	E-mail	Telefone	Tipo
				Financiamento

Palavra Chave

Palavra-chave
Fascite Plantar
Ácido Hialurônico
Tratamento por Ondas de Choque Extracorpóreas

Detalhamento do Estudo

Resumo:

A fascite plantar é uma afecção extremamente comum no dia a dia dos ortopedistas. Existem inúmeros tratamentos não invasivos descritos, porém em alguns casos não surgem efeito e podem evoluir para a cronicidade da lesão. Assim, alguns métodos mais invasivos como a infiltração da fáscia plantar com ácido hialurônico. O ácido hialurônico é muito utilizado nas artroses do joelho e outras articulações como opção para retardar o tratamento cirúrgico e como adjuvante no tratamento conservador. As propriedades bioquímicas dessa substância garantem uma diminuição do processo inflamatório bem como nutre a cartilagem articular. Desse modo, a infiltração do hialuronato de sódio pode atuar como mediador inflamatório e analgésico, evitando as complicações comuns causadas pela infiltração com corticosteroides. Outra opção para o tratamento da fascite plantar crônica é a terapia por ondas de choque, com diversos estudos demonstrando a sua eficácia. O objetivo desse estudo é comparar o efeito terapêutico da aplicação única de hialuronato de sódio para a fascite plantar crônica guiada por ultrassom com a terapia por ondas de choque.

Introdução:

O uso do hialuronato de sódio apresenta-se como importante tratamento não cirúrgico nas artroses do joelho, sendo uma opção no tratamento conservador. Essa substância possui características como mediadora da inflamação, reduzindo a passagem dessas células até o local aplicado.3-5

O uso do ácido hialurônico também foi descrito para o tratamento das patologias relacionados as inflamações extra-articulares, como tendões, ligamentos e fáscias. O estresse excessivo pode se concentrar na interface entre estruturas com propriedades mecânicas diferentes, causando uma variedade de condições patológicas.6

O tratamento mais utilizado para as entesopatias e especificamente a fascite plantar incluem alongamento de cadeia posterior, fisioterapia e uso drogas anti-inflamatórias não esteroidais.5-7 Outra opção para o tratamento da fascite plantar crônica é a terapia por ondas de choque, com diversos estudos demonstrando a sua eficácia.8-11

O objetivo do estudo é comparar a eficácia terapêutica da aplicação de hialuronato de sódio para a fascite plantar crônica guiada por ultrassom com a terapia por ondas de choque.

Hipótese:

Acredita-se que o hialuronato de sódio possa permitir uma melhor reabilitação fisioterápica com menor quadro álgico e maior mobilidade, evitando procedimentos cirúrgicos custosos e possíveis riscos de complicações.

Objetivo Primário:

O objetivo primário do estudo é avaliar o efeito analgésico e anti-inflamatório da aplicação única de hialuronato de sódio para a fascite plantar crônica e comparar com a terapia por ondas de choque.

Objetivo Secundário:

O objetivo secundário do estudo é avaliar a função do pé e critérios de satisfação após a infiltração única de hialuronato de sódio para a fascite plantar crônica e comparar com a terapia por ondas de choque.

Metodologia Proposta:

Serão incluídos no estudo os pacientes com diagnóstico de fascite plantar nos ambulatórios da unidade de ortopedia e traumatologia do Hospital Sancta Maggiore Mooca que não apresentaram melhora dos sintomas com três meses no mínimo de tratamento conservador.

Os pacientes serão convidados a participar do estudo, e apresentados o termo de consentimento livre esclarecido (TCLE). Somente serão incluídos os indivíduos que assinarem o termo e concordar com os métodos de avaliação.

Após isso, tais pacientes serão randomizados para o grupo da infiltração com ácido hialurônico ou no grupo da terapia por ondas de choque. Os tratamentos serão realizados no ambulatório do próprio hospital. A aplicação do ácido hilurônico será realizada pelo mesmo profissional de saúde. Já o tratamento por ondas de choque, será realizada por outro profissional de saúde. A avaliação clínica com aplicação dos escores de dor, função e satisfação pessoal será realizado durante 24 meses nos seguintes intervalos de consultas: 1, 3, 6, 12 e 24.

O tamanho da amostra foi calculado através do pacote “pwr” do software R com previsão para o “teste T” segundo os parâmetros: teste do poder de 0,75, tamanho do efeito de 0,3 (proposto por Cohen) e nível de significância de 0,05, sendo o considerado necessário 100 participantes.

Desse modo, conseguiremos avaliar o resultado no curto, médio e longo prazo. Associado as terapias, os pacientes permanecerão em trabalho de reabilitação com fisioterapia. Os dados serão documentados no programa Excel, e, em seguida, realizada análise dos dados. O trabalho será submetido à Comissão de Ética local de acordo com a orientação da Plataforma Brasil.

Não haverá nenhum custo para a operadora de saúde ou para o próprio paciente nessa avaliação visto que ela será realizada em consultas de rotina previamente agendadas no ambulatório do autor e dos coautores.

A randomização será determinada usando uma tabela em bloco de randomização gerada por computador. A randomização em bloco (tamanhos aleatórios de 2, 4, 6 e 8) será usada para garantir que os grupos de tratamento sejam equilibrados ao longo do período do estudo. Essa estratégia garante que grupo intervenção e grupo controle sejam equilibrados quanto ao número de participantes. Para garantir o sigilo de alocação, deve-se utilizar variação aleatória dos tamanhos dos blocos (quatro a oito participantes por bloco). Utilizaremos o pacote blockrand para o software R para gerar a tabela de randomização.

Riscos:

A infiltração local do hialuronato de sódio pode ocorrer efeitos adversos como dor, sensação de calor, vermelhidão e edema, descritos na bula do medicamento. Não há riscos relacionados a drogas, exposição a agentes tóxicos, radioativos e medicamentos não autorizados pelas agências nacionais reguladoras. A terapia por ondas de choque poderá provocar um processo inflamatório e dor

local, mas em geral é rapidamente resolvida com analgésicos leves.

Benefícios:

A infiltração da fáscia plantar com o hialuronato de sódio apresenta vantagens descritas como no controle da dor, reposição do líquido peritendíneo, podendo proporcionar conforto prolongado e melhor reabilitação. A terapia por ondas de choque apresenta-se com excelente benefício para a fascite plantar como já descrito na literatura.

Metodologia de Análise de Dados:

As associações serão testadas pelo teste comparativo de médias de Mann-Whitney com nível de significância de 5%, após avaliar a distribuição das amostras pelo teste de Kolmogorov-Smirnov, e, portanto, consideradas associações estatisticamente significativas aquelas cujo valor de p foi inferior a 0,05. Utilizaremos o teste de chi-quadrado ou exato de Fischer para avaliar as variáveis categóricas. A análise estatística será realizada através do pacote stats para o software R.

Desfecho Primário:

Essa técnica poderá ser usada nas fascites plantares crônicas como coadjuvante para analgesia e melhor reabilitação, evitando procedimento cirúrgicos invasivos e custosos.

Tamanho da Amostra no Brasil: 100

Data do Primeiro Recrutamento: 02/03/2020

Países de Recrutamento

País de Origem do Estudo	País	Nº de participantes da pesquisa
Sim	BRASIL	100

Outras Informações

Haverá uso de fontes secundárias de dados (prontuários, dados demográficos, etc)?

Não

Informe o número de indivíduos abordados pessoalmente, recrutados, ou que sofrerão algum tipo de intervenção neste centro de pesquisa:

100

Grupos em que serão divididos os participantes da pesquisa neste centro

ID Grupo	Nº de Indivíduos	Intervenções a serem realizadas
Ácido Hialurônico	50	Infiltração da fáscia plantar guiada por ultrassom com ácido hialurônico
Tratamento por Ondas de Choque Extracorpóreas	50	Três sessões de terapia por ondas de choque extracorpórea

O Estudo é Multicêntrico no Brasil?

Não

Propõe dispensa do TCLE?

Não

Haverá retenção de amostras para armazenamento em banco?

Não

Cronograma de Execução

Identificação da Etapa	Início (DD/MM/AAAA)	Término (DD/MM/AAAA)
Envio do Projeto a Comissão de ética	01/11/2019	01/12/2019
Elaboração do projeto	23/10/2019	30/10/2019
Análise dos resultados e correção estatística	01/02/2021	28/02/2021
Idealização do tema	23/10/2019	30/10/2019
Elaboração do artigo para publicação	01/03/2021	01/05/2021
Envio do artigo para publicação	01/06/2021	01/07/2021
Coleta de dados	01/03/2020	01/01/2021

Orçamento Financeiro

Identificação de Orçamento	Tipo	Valor em Reais (R$)
Papelaria	Custeio	R$ 200,00
Total em R$		R$ 200,00

Bibliografia:

A pesquisa bibliográfica foi realizada através dos bancos de dados PubMed, Bireme e livros- texto referentes ao tema estudado. 1. Downie W, Leatham P, Rhind V, Wright V, Branco J, Anderson J. Studies with pain rating scales. Ann Rheum Dis 1978;37(4):378-81. 2. Coughlin MJ. Treatment of bunionette deformity with longitudinal diaphyseal osteotomy with distal soft tissue repair. Foot Ankle 1991;11(4):195-203. 3. Kitaoka HB, Alexander IJ, Adelaar RS, Nunley JA, Myerson MS, Sanders M. Clinical rating systems for the ankle-hindfoot, midfoot, hallux, and lesser toes. Foot Ankle Int 1994;15(7):349-53. doi: 10.1177/107110079401500701. 4. Altman R, Lim S, Steen RG, Dasa V. Hyaluronic acid injections are associated with delay of total knee replacement surgery in patients with knee osteoarthritis: evidence from a large U.S. health claims database. PLoS One 10:e0145776, 2015. 5. Saw KY, Hussin P, Loke SC, Azam M, Chen HC, Tay YG, Low S, Wallin KL, Ragavanaidu K. Articular cartilage regeneration with autologous marrow aspirate and hyaluronic acid: an experimental study in a goat model. Arthroscopy 25:1391–1400, 2009. 6. Petrella RJ, Petrella MJ, Cogliano A. Periarticular hyaluronic acid in acute ankle sprain. Clin J Sport Med 17:251–257, 2007. 7. Petrella MJ, Cogliano A, Petrella RJ. Original research: long-term efficacy and safety of periarticular hyaluronic acid in acute ankle sprain. Phys Sportsmed 37:64–70,2009. 8. Böddeker R, Schäfer H, Haake M (2001) Extracorporeal shockwave therapy (ESWT) in the treatment of plantar fasciitis–a biometrical review. Clin Rheumatol 20(5):324–330 9. Buchbinder R, Ptasznik R, Gordon J et al (2002) Ultrasoundguided extracorporeal shock wave therapy for plantar fasciitis: a randomized controlled trial. JAMA 288(11):1364–1372 10. Basford JR, Malanga GA, Krause DA et al (1998) A randomized controlled evaluation of low-intensity laser therapy: plantar fasciitis. Arch Phys Med Rehabil 79(3):249–254 11. Kamel M, Kotob H (2000) High frequency ultrasonographic findings in plantar fasciitis and assessment of local steroid injection. J Rheumatol 27(9):2139–2141

Upload de Documentos

Arquivo Anexos:

Tipo	Arquivo
Projeto Detalhado / Brochura Investigador	PROJETO_PESQUISA.pdf
Declaração de Pesquisadores	Declaracao_do_Pesquisador.pdf
Cronograma	CRONOGRAMA.pdf
Outros	Parecer.pdf
Folha de Rosto	FolhaDeRosto.pdf
TCLE / Termos de Assentimento / Justificativa de Ausência	TCLE.pdf
Outros	APRESENTACAO.pdf
Declaração de Instituição e Infraestrutura	infraestrutura.pdf
Orçamento	ORCAMENTO.pdf

Finalizar

Manter sigilo da integra do projeto de pesquisa: Não
