# Supplementary material for: Comparison of the effect of hyaluronic acid injection versus extracorporeal shockwave therapy on chronic plantar fasciitis: Protocol for a randomized controlled trial
Source: PLoS One. 2021 Jun 24;16(6):e0250768. doi: 10.1371/journal.pone.0250768 (PMC8224905; doi:10.1371/journal.pone.0250768)
Supplement: S3 File — (PDF) [file pone.0250768.s004.pdf]

CONSUBSTANCED OPINION OF THE CEP

AMENDMENT DATA

Research Title: A comparação do efeito da injeção de ácido hialurônico versus terapia por ondas de choque extracorpórea na fascite plantar crônica: ensaio clínico randomizado

Pesquisador: GABRIEL FERRAZ FERREIRA

Área Temática:

Versão: 4

CAAE: 25585319.5.0000.8114

Instituição Proponente: PREVENT SENIOR PRIVATE OPERADORA DE SAUDE LTDA

Main Sponsor: Own Financing

OPINION DATA

Opinion Number: 4,227,201

Project presentation:

The present study will be a prospective randomized controlled clinical trial, whose treatment effectiveness will be evaluated by comparing two distinct groups: treatment group that will receive sodium hyaluronate and the shock wave treatment group. Patients included in the study will come from the outpatient clinic of the Orthopedics and Traumatology Unit of Hospital Sancta Maggiore Mooca. The objective of the study is compare pain outcomes: visual analog scale (VAS) 1, mobility function (AOFAS) 2, and determine the effectiveness of these treatments. Plantar fasciitis is an extremely common condition in orthopedists' daily lives. There are numerous described non-invasive treatments, however in some cases, there is no effect and may evolve to chronicity of the lesion. Thus, some more invasive methods such as infiltration of the fascia plant with hyaluronic acid. Hyaluronic acid is widely used in arthrosis of the knee and other joints as an option to delay the surgical treatment and as an adjunct to conservative treatment. The biochemical properties of this substance guarantee a decrease in the inflammatory process as well as nourishes the articular cartilage. Thus, the infiltration of sodium hyaluronate can act as a mediator

inflammatory and analgesic, avoiding the common complications caused by infiltration with corticosteroids. Another option for the treatment of chronic plantar fasciitis is shockwave therapy, with several studies demonstrating its effectiveness. The objective of this study is to compare the therapeutic effect of the single application of sodium hyaluronate for chronic plantar fasciitis guided by ultrasound with shock wave therapy.

Research Objective:
Primary Objective:
The primary objective of the study is to assess the analgesic and anti-inflammatory effect of the single application of sodium hyaluronate for chronic plantar fasciitis and to compare it with shockwave therapy.

Secondary Objective:
The secondary objective of the study is to assess foot function and satisfaction criteria after a single sodium hyaluronate infiltration for chronic plantar fasciitis and to compare it with shock wave therapy.

Assessment of Risks and Benefits:
Local infiltration of sodium hyaluronate can cause adverse effects such as pain, a sensation of heat, redness and edema, as described in the package insert. There are no risks related to drugs, exposure to toxic, radioactive agents and drugs not authorized by national regulatory agencies. Shockwave therapy may cause an inflammatory process and local pain, but it is usually quickly resolved with mild pain relievers.

Benefits:
The infiltration of the plantar fascia with sodium hyaluronate presents advantages described as in pain control, replacement of peritendinous fluid, which can provide prolonged comfort and better rehabilitation. Shock wave therapy has an excellent benefit for plantar fasciitis as described in the literature.

Research Comments and Considerations:
Amendment presented to change the number of participants with a reduction from 100 to 80,

Endereço: Rua Lourenço Marques, 158, 9º andar, sala CEP
Bairro: Vila Olímpia **CEP:** 04.547-100
UF: SP **Município:** SAO PAULO
Telefone: (11)4085-9070 **E-mail:** cepps@preventsenior.com.br

maintaining the quality of the data to be obtained.

The sample size was calculated using the “pwr” package of software R with a prediction for the “T test” according to the parameters: power test of 0.80, effect size of 0.3 (proposed by Cohen) and level of significance of 0.001, with the one considered
80 participants required.

Mandatory submission terms considerations: not applicable

Recommendations: not applicable

Conclusions or Pending and List of Inadequacies:
The project entitled "Comparison of the effect of hyaluronic acid injection versus extracorporeal shock wave therapy in chronic plantar fasciitis: a randomized clinical trial" had its amendment approved.

Final Considerations at the discretion of the CEP:
This opinion was prepared based on the documents listed below:

Tipo Documento	Arquivo	Postagem	Autor	Situação
Informações Básicas do Projeto	PB_INFORMAÇÕES_BÁSICAS_1613401_E1.pdf	20/08/2020 15:11:31		Aceito
Orçamento	ORCAMENTO_TERCEIRA_REVISAO.pdf	20/08/2020 15:06:54	GABRIEL FERRAZ FERREIRA	Aceito
Projeto Detalhado / Brochura Investigador	PROJETO_PESQUISA_SEXTA_REVISAO.pdf	20/08/2020 15:05:36	GABRIEL FERRAZ FERREIRA	Aceito
Outros	CARTA_RESPOSTA_SEXTA_REVISAO.pdf	20/02/2020 15:00:52	GABRIEL FERRAZ FERREIRA	Aceito
Outros	Termo_Confidencialidade.pdf	20/02/2020 14:59:57	GABRIEL FERRAZ FERREIRA	Aceito
TCLE / Termos de Assentimento / Justificativa de Ausência	TCLE_QUINTA_REVISAO.pdf	20/02/2020 14:59:08	GABRIEL FERRAZ FERREIRA	Aceito
Outros	Validacao_Gabriel07.pdf	31/01/2020 14:45:09	DANIELA RIMOLDI CUNHA	Aceito
Outros	Validacao_Gabriel06.pdf	24/01/2020 16:40:15	DANIELA RIMOLDI CUNHA	Aceito

Outros	Validacao_Gabriel_05.docx	13/01/2020 15:34:52	DANIELA RIMOLDI CUNHA	Aceito
Outros	CARTA_CEP_29_12_19.pdf	29/12/2019 21:40:11	GABRIEL FERRAZ FERREIRA	Aceito
Outros	Validacao_gabriel04.pdf	20/12/2019 11:09:04	Henrique Guindalini Deliberato	Aceito
Outros	Parecer_IPS.pdf	18/12/2019 17:46:42	GABRIEL FERRAZ FERREIRA	Aceito
Folha de Rosto	FOLHA_DE_ROSTO.pdf	18/12/2019 17:45:02	GABRIEL FERRAZ FERREIRA	Aceito
Outros	Validacao_Gabriel03.pdf	02/12/2019 15:04:56	Henrique Guindalini Deliberato	Aceito
Outros	Validacao_Gabriel02.pdf	11/11/2019 13:58:23	Henrique Guindalini Deliberato	Aceito
Outros	Validacao_Gabriel01.pdf	04/11/2019 16:21:02	Henrique Guindalini Deliberato	Aceito
Declaração de Instituição e Infraestrutura	infraestrutura.pdf	01/11/2019 11:54:02	GABRIEL FERRAZ FERREIRA	Aceito
Outros	APRESENTACAO.pdf	23/10/2019 11:32:00	GABRIEL FERRAZ FERREIRA	Aceito
Declaração de Pesquisadores	Declaracao_do_Pesquisador.pdf	23/10/2019 11:31:31	GABRIEL FERRAZ FERREIRA	Aceito

Situação do Parecer:
Aprovado

Necessita Apreciação da CONEP:
Não

SAO PAULO, 20 de Agosto de 2020

Assinado por:
PATRICIA ESPINDOLA BRETAS BERBARE
(Coordenador(a))
