# Supplementary material for: Comparison of the effect of hyaluronic acid injection versus extracorporeal shockwave therapy on chronic plantar fasciitis: Protocol for a randomized controlled trial
Source: PLoS One. 2021 Jun 24;16(6):e0250768. doi: 10.1371/journal.pone.0250768 (PMC8224905; doi:10.1371/journal.pone.0250768)
Supplement: S4 File — (PDF) [file pone.0250768.s005.pdf]

PARECER CONSUBSTANCIADO DO CEP

DADOS DA EMENDA

Título da Pesquisa: A comparação do efeito da injeção de ácido hialurônico versus terapia por ondas de choque extracorpórea na fascite plantar crônica: ensaio clínico randomizado

Pesquisador: GABRIEL FERRAZ FERREIRA
Área Temática:
Versão: 4
CAAE: 25585319.5.0000.8114
Instituição Proponente: PREVENT SENIOR PRIVATE OPERADORA DE SAUDE LTDA
Patrocinador Principal: Financiamento Próprio

DADOS DO PARECER

Número do Parecer: 4.227.201

Apresentação do Projeto:
O presente estudo será um ensaio clínico randomizado controlado, prospectivo, cuja eficácia do tratamento será avaliada por comparação entre dois
grupos distintos: grupo de tratamento que receberá hialuronato de sódio e o grupo de tratamento por ondas de choque. Os pacientes incluídos no
estudo serão provenientes do ambulatório da Unidade de ortopedia e traumatologia do Hospital Sancta Maggiore Mooca. O Objetivo do estudo é
comparar os desfechos de dor: escala visual analógica (EVA)1, função mobilidade (AOFAS)2, e determinar a eficácia desses tratamentos. A fascite plantar é uma afecção extremamente comum no dia a dia dos ortopedistas. Existem inúmeros tratamentos não invasivos descritos, porém
em alguns casos não surgem efeito e podem evoluir para a cronicidade da lesão. Assim, alguns métodos mais invasivos como a infiltração da fáscia
plantar com ácido hialurônico. O ácido hialurônico é muito utilizado nas artroses do joelho e outras articulações como opção para retardar o
tratamento cirúrgico e como adjuvante no tratamento conservador. As propriedades bioquímicas dessa substância garantem uma diminuição do
processo inflamatório bem como nutre a cartilagem articular. Desse modo, a infiltração do hialuronato de sódio pode atuar como mediador

inflamatório e analgésico, evitando as complicações comuns causadas pela infiltração com corticosteroides. Outra opção para o tratamento da
fascite plantar crônica é a terapia por ondas de choque, com diversos estudos demonstrando a sua eficácia. O objetivo desse estudo é comparar o
efeito terapêutico da aplicação única de hialuronato de sódio para a fascite plantar crônica guiada por ultrassom com a terapia por ondas de choque.

Objetivo da Pesquisa:

Objetivo Primário:
O objetivo primário do estudo é avaliar o efeito analgésico e anti-inflamatório da aplicação única de hialuronato de sódio para a fascite plantar
crônica e comparar com a terapia por ondas de choque.
Objetivo Secundário:
O objetivo secundário do estudo é avaliar a função do pé e critérios de satisfação após a infiltração única de hialuronato de sódio para a fascite
plantar crônica e comparar com a terapia por ondas de choque.

Avaliação dos Riscos e Benefícios:

A infiltração local do hialuronato de sódio pode ocorrer efeitos adversos como dor, sensação de calor, vermelhidão e edema, descritos na bula do
medicamento. Não há riscos relacionados a drogas, exposição a agentes tóxicos, radioativos e medicamentos não autorizados pelas agências
nacionais reguladoras. A terapia por ondas de choque poderá provocar um processo inflamatório e dor local, mas em geral é rapidamente resolvida
com analgésicos leves.
Benefícios:
A infiltração da fáscia plantar com o hialuronato de sódio apresenta vantagens descritas como no controle da dor, reposição do líquido peritendíneo,
podendo proporcionar conforto prolongado e melhor reabilitação. A terapia por ondas de choque apresenta-se com excelente benefício para a fascite
plantar como já descrito na literatura.

Comentários e Considerações sobre a Pesquisa:

Emenda apresentada para alteração do número de participantes com redução de 100 para 80,

mantendo a qualidade dos dados que serão obtidos.
O tamanho da amostra foi calculado através do pacote “pwr” do software R com previsão para o “teste T” segundo os
parâmetros: teste do poder de 0,80, tamanho do efeito de 0,3 (proposto por Cohen) e nível de significância de 0,001, sendo o considerado
necessário 80 participantes.

Considerações sobre os Termos de apresentação obrigatória:

não se aplica

Recomendações:

não se aplica

Conclusões ou Pendências e Lista de Inadequações:

O projeto entitulado "A comparação do efeito da injeção de ácido hialurônico versus terapia por ondas de choque extracorpórea na fascite plantar crônica: ensaio clínico randomizado" teve sua emenda aprovada.

Considerações Finais a critério do CEP:

Este parecer foi elaborado baseado nos documentos abaixo relacionados:

Tipo Documento	Arquivo	Postagem	Autor	Situação
Informações Básicas do Projeto	PB_INFORMAÇÕES_BÁSICAS_1613401_E1.pdf	20/08/2020 15:11:31		Aceito
Orçamento	ORCAMENTO_TERCEIRA_REVISAO.pdf	20/08/2020 15:06:54	GABRIEL FERRAZ FERREIRA	Aceito
Projeto Detalhado / Brochura Investigador	PROJETO_PESQUISA_SEXTA_REVISAO.pdf	20/08/2020 15:05:36	GABRIEL FERRAZ FERREIRA	Aceito
Outros	CARTA_RESPOSTA_SEXTA_REVISAO.pdf	20/02/2020 15:00:52	GABRIEL FERRAZ FERREIRA	Aceito
Outros	Termo_Confidencialidade.pdf	20/02/2020 14:59:57	GABRIEL FERRAZ FERREIRA	Aceito
TCLE / Termos de Assentimento / Justificativa de Ausência	TCLE_QUINTA_REVISAO.pdf	20/02/2020 14:59:08	GABRIEL FERRAZ FERREIRA	Aceito
Outros	Validacao_Gabriel07.pdf	31/01/2020 14:45:09	DANIELA RIMOLDI CUNHA	Aceito
Outros	Validacao_Gabriel06.pdf	24/01/2020 16:40:15	DANIELA RIMOLDI CUNHA	Aceito

Outros	Validacao_Gabriel_05.docx	13/01/2020 15:34:52	DANIELA RIMOLDI CUNHA	Aceito
Outros	CARTA_CEP_29_12_19.pdf	29/12/2019 21:40:11	GABRIEL FERRAZ FERREIRA	Aceito
Outros	Validacao_gabriel04.pdf	20/12/2019 11:09:04	Henrique Guindalini Deliberato	Aceito
Outros	Parecer_IPS.pdf	18/12/2019 17:46:42	GABRIEL FERRAZ FERREIRA	Aceito
Folha de Rosto	FOLHA_DE_ROSTO.pdf	18/12/2019 17:45:02	GABRIEL FERRAZ FERREIRA	Aceito
Outros	Validacao_Gabriel03.pdf	02/12/2019 15:04:56	Henrique Guindalini Deliberato	Aceito
Outros	Validacao_Gabriel02.pdf	11/11/2019 13:58:23	Henrique Guindalini Deliberato	Aceito
Outros	Validacao_Gabriel01.pdf	04/11/2019 16:21:02	Henrique Guindalini Deliberato	Aceito
Declaração de Instituição e Infraestrutura	infraestrutura.pdf	01/11/2019 11:54:02	GABRIEL FERRAZ FERREIRA	Aceito
Outros	APRESENTACAO.pdf	23/10/2019 11:32:00	GABRIEL FERRAZ FERREIRA	Aceito
Declaração de Pesquisadores	Declaracao_do_Pesquisador.pdf	23/10/2019 11:31:31	GABRIEL FERRAZ FERREIRA	Aceito

Situação do Parecer:
Aprovado

Necessita Apreciação da CONEP:
Não

SAO PAULO, 20 de Agosto de 2020

Assinado por:
PATRICIA ESPINDOLA BRETAS BERBARE
(Coordenador(a))

Endereço: Rua Lourenço Marques, 158, 9º andar, sala CEP
Bairro: Vila Olímpia **CEP:** 04.547-100
UF: SP **Município:** SAO PAULO
Telefone: (11)4085-9070 **E-mail:** cepps@preventsenior.com.br
