# Supplementary material for: Comparison of the effect of hyaluronic acid injection versus extracorporeal shockwave therapy on chronic plantar fasciitis: Protocol for a randomized controlled trial
Source: PLoS One. 2021 Jun 24;16(6):e0250768. doi: 10.1371/journal.pone.0250768 (PMC8224905; doi:10.1371/journal.pone.0250768)
Supplement: S5 File — (PDF) [file pone.0250768.s006.pdf]

PREVENT SENIOR

CONSUBSTANCED OPINION OF THE CEP

RESEARCH PROJECT DATA

Search Title: Comparison of the effect of hyaluronic acid injection versus extracorporeal shockwave therapy on chronic plantar fasciitis: Protocol for a randomized controlled trial

Researcher: GABRIEL FERRAZ FERREIRA

Thematic Area:

Version: 2

CAAE: 25585319.5.0000.8114

Proponent Institution: PREVENT SENIOR PRIVATE OPERADORA DE SAUDE LTDA

Main Sponsor: Own Financing

OPINION DATA

Opinion Number: 3,912,441

Project presentation:

Plantar fasciitis is an extremely common condition in orthopedists' daily lives.
There are countless non-invasive treatments described, but in some cases they do not show any effect and can evolve to the chronicity of the lesion. Thus, some more invasive methods such as infiltration of the plantar fascia with hyaluronic acid. Hyaluronic acid is widely used in arthrosis of the knee and other joints as an option to delay surgical treatment and as an adjunct to conservative treatment.
The biochemical properties of this substance guarantee a decrease in the inflammatory process as well as nourishing the articular cartilage. Thus, sodium hyaluronate infiltration can act as an inflammatory and analgesic mediator, avoiding the common complications caused by infiltration with corticosteroids. Another option for the treatment of chronic plantar fasciitis is shockwave therapy, with several studies demonstrating its effectiveness.
The present study will be a prospective randomized controlled clinical trial, whose treatment effectiveness will be evaluated by comparing two different groups: treatment group that will receive sodium hyaluronate and the shock wave treatment group. The patients included in the study will come from the outpatient clinic of the Orthopedics and

traumatologist Hospital Sancta Maggiore Mooca. The objective of the study is to compare pain outcomes: visual analog scale (VAS), mobility function (AOFAS), and determine the effectiveness of these treatments.

Research Objective:
PRIMARY OBJECTIVE:

The primary objective of the study is to evaluate the analgesic and anti-inflammatory effect of the single application of sodium hyaluronate for chronic plantar fasciitis and to compare it with shock wave therapy.

SECONDARY OBJECTIVE:
The secondary objective of the study is to assess foot function and satisfaction criteria after a single infiltration of sodium hyaluronate for chronic plantar fasciitis and to compare it with shock wave therapy.

Assessment of Risks and Benefits:

RISKS:
The local infiltration of sodium hyaluronate can cause adverse effects such as pain, feeling of heat, redness and edema, described in the package insert. There are no risks related to drugs, exposure to toxic, radioactive agents and drugs not authorized by national regulatory agencies. Shockwave therapy may cause an inflammatory process and local pain, but it is usually quickly resolved with mild pain relievers.

BENEFITS:
The infiltration of the plantar fascia with sodium hyaluronate has advantages described as in pain control, replacement of peritendinous fluid, which can provide prolonged comfort and better rehabilitation. Shock wave therapy has an excellent benefit for plantar fasciitis as described in the literature.

Research Comments and Considerations:

The study is relevant to scientific medical development in general, as it aims to analyze the use of less invasive products and procedures to the detriment of more costly procedures and high rehabilitation of the patient.

Thus, such a study can be a clinical reference for future interventions in the field of orthopedics

Mandatory submission terms considerations:

All terms were changed as requested in a previous opinion and are in accordance with CEP requirements and guidelines.

Recommendations:

Attention is recommended in compliance with the requirements of Res. 466/12 and complementary and Guide to Good Practices.

Pay attention to the period and send partial reports (half-yearly) and final study report to the CEP-CONEP system, via the Brazil platform, to monitor the development of the work.

Conclusions or Pending and List of Inadequacies:

The pending issues were met and / or justified.

Final Considerations at the discretion of the CEP:

This opinion was prepared based on the documents listed below:

Document Type	File	Post	Author	Situation
Basic Project Information	PB_INFORMAÇÕES_BÁSICAS_DO_PROJETO_1458201.pdf	20/02/2020 15:01:35		OK
Budget	ORCAMENTO_SEGUNDA_REVISAO.pdf	20/02/2020 15:01:22	GABRIEL FERRAZ FERREIRA	OK
Detailed Project / Researcher Brochure	PROJETO_PESQUISA_QUINTA_REVISAO.pdf	20/02/2020 15:01:12	GABRIEL FERRAZ FERREIRA	OK
Others	CARTA_RESPOSTA_SEXTA_REVISAO.pdf	20/02/2020 15:00:52	GABRIEL FERRAZ FERREIRA	OK
Schedule	CRONOGRAMA_PRIMEIRA_REVISAO.pdf	20/02/2020 15:00:11	GABRIEL FERRAZ FERREIRA	OK
Others	Termo_Confidencialidade.pdf	20/02/2020 14:59:57	GABRIEL FERRAZ FERREIRA	OK
IC / Terms of	TCLE_QUINTA_REVISAO.pdf	20/02/2020	GABRIEL FERRAZ	OK

Assentimento / Justificativa de Ausência	TCLE_QUINTA_REVISAO.pdf	14:59:08	FERREIRA	Aceito
Outros	Validacao_Gabriel07.pdf	31/01/2020 14:45:09	DANIELA RIMOLDI CUNHA	Aceito
Outros	Validacao_Gabriel06.pdf	24/01/2020 16:40:15	DANIELA RIMOLDI CUNHA	Aceito
Outros	Validacao_Gabriel_05.docx	13/01/2020 15:34:52	DANIELA RIMOLDI CUNHA	Aceito
Outros	CARTA_CEP_29_12_19.pdf	29/12/2019 21:40:11	GABRIEL FERRAZ FERREIRA	Aceito
Outros	Validacao_gabriel04.pdf	20/12/2019 11:09:04	Henrique Guindalini Deliberato	Aceito
Outros	Parecer_IPS.pdf	18/12/2019 17:46:42	GABRIEL FERRAZ FERREIRA	Aceito
Folha de Rosto	FOLHA_DE_ROSTO.pdf	18/12/2019 17:45:02	GABRIEL FERRAZ FERREIRA	Aceito
Outros	Validacao_Gabriel03.pdf	02/12/2019 15:04:56	Henrique Guindalini Deliberato	Aceito
Outros	Validacao_Gabriel02.pdf	11/11/2019 13:58:23	Henrique Guindalini Deliberato	Aceito
Outros	Validacao_Gabriel01.pdf	04/11/2019 16:21:02	Henrique Guindalini Deliberato	Aceito
Declaração de Instituição e Infraestrutura	infraestrutura.pdf	01/11/2019 11:54:02	GABRIEL FERRAZ FERREIRA	Aceito
Outros	APRESENTACAO.pdf	23/10/2019 11:32:00	GABRIEL FERRAZ FERREIRA	Aceito
Declaração de Pesquisadores	Declaracao_do_Pesquisador.pdf	23/10/2019 11:31:31	GABRIEL FERRAZ FERREIRA	Aceito

Opinion Status:
Approved

Needs CONEP Appreciation:
No

SAO PAULO, 12 de Março de 2020

Assinado por:
PATRICIA ESPINDOLA BRETAS BERBARE
(Coordenador(a))

Endereço: Rua Lourenço Marques 158, 6º andar
Bairro: VILA OLIMPIA **CEP:** 04.550-004
UF: SP **Município:** SAO PAULO
Telefone: (11)4085-9070 **E-mail:** cepps@preventsenior.com.br
