# Supplementary material for: Comparison of the effect of hyaluronic acid injection versus extracorporeal shockwave therapy on chronic plantar fasciitis: Protocol for a randomized controlled trial
Source: PLoS One. 2021 Jun 24;16(6):e0250768. doi: 10.1371/journal.pone.0250768 (PMC8224905; doi:10.1371/journal.pone.0250768)
Supplement: S6 File — (PDF) [file pone.0250768.s007.pdf]

PREVENT SENIOR

PARECER CONSUBSTANCIADO DO CEP

DADOS DO PROJETO DE PESQUISA

Título da Pesquisa: A comparação do efeito da injeção de ácido hialurônico versus terapia por ondas de choque extracorpórea na fascite plantar crônica: ensaio clínico randomizado

Pesquisador: GABRIEL FERRAZ FERREIRA
Área Temática:
Versão: 2
CAAE: 25585319.5.0000.8114
Instituição Proponente: PREVENT SENIOR PRIVATE OPERADORA DE SAUDE LTDA
Patrocinador Principal: Financiamento Próprio

DADOS DO PARECER

Número do Parecer: 3.912.441

Apresentação do Projeto:
A fascite plantar é uma afecção extremamente comum no dia a dia dos ortopedistas.
Existem inúmeros tratamentos não invasivos descritos, porém em alguns casos não surgem efeito e podem evoluir para a cronicidade da lesão. Assim, alguns métodos mais invasivos como a infiltração da fáscia plantar com ácido hialurônico. O ácido hialurônico é muito utilizado nas artroses do joelho e outras articulações como opção para retardar o tratamento cirúrgico e como adjuvante no tratamento conservador.

As propriedades bioquímicas dessa substância garantem uma diminuição do processo inflamatório bem como nutre a cartilagem articular. Desse modo, a infiltração do hialuronato de sódio pode atuar como mediador inflamatório e analgésico, evitando as complicações comuns causadas pela infiltração com corticosteroides. Outra opção para o tratamento da fascite plantar crônica é a terapia por ondas de choque, com diversos estudos demonstrando a sua eficácia.

O presente estudo será um ensaio clínico randomizado controlado,prospectivo, cuja eficácia do tratamento será avaliada por comparação entre dois grupos distintos: grupo de tratamento que receberá hialuronato de sódio e o grupo de tratamento por ondas de choque. Os pacientes incluídos no estudo serão provenientes do ambulatório da Unidade de ortopedia e

Endereço: Rua Lourenço Marques 158, 6º andar
Bairro: VILA OLIMPIA **CEP:** 04.550-004
UF: SP **Município:** SAO PAULO
Telefone: (11)4085-9070 **E-mail:** cepps@preventsenior.com.br

traumatologiado Hospital Sancta Maggiore Mooca. O Objetivo do estudo é comparar os desfechos de dor: escala visual analógica (EVA), função mobilidade (AOFAS), e determinar a eficácia desses tratamentos.

Objetivo da Pesquisa:
OBJETIVO PRIMÁRIO:

O objetivo primário do estudo é avaliar o efeito analgésico e anti-inflamatório da aplicação única de hialuronato de sódio para a fascite plantar crônica e comparar com
a terapia por ondas de choque.

OBJETIVO SECUNDÁRIO:

O objetivo secundário do estudo é avaliar a função do pé e critérios de satisfação após a infiltração única de hialuronato de sódio para a fascite plantar crônica e comparar com a terapia por ondas de choque.

Avaliação dos Riscos e Benefícios:
RISCOS:

A infiltração local do hialuronato de sódio pode ocorrer efeitos adversos como dor, sensação de calor, vermelhidão e edema, descritos na bula do medicamento. Não há riscos relacionados a drogas, exposição a agentes tóxicos, radioativos e medicamentos não autorizados pelas agências nacionais reguladoras. A terapia por ondas de choque poderá provocar um processo inflamatório e dor local, mas em geral é rapidamente resolvida com analgésicos leves.

BENEFÍCIOS:

A infiltração da fáscia plantar com o hialuronato de sódio apresenta vantagens descritas como no controle da dor, reposição do líquido peritendíneo, podendo proporcionar conforto prolongado e melhor reabilitação. A terapia por ondas de choque apresenta-se com excelente benefício para a fascite plantar como já descrito na literatura.

Comentários e Considerações sobre a Pesquisa:

O estudo apresenta relevância para a o desenvolvimento médico científico de modo geral uma vez que visa analisar a utilização de produtos e procedimentos menos invasivos em detrimento de procedimentos mais custosos e de elevada reabilitação do paciente. Assim, tal estudo pode ser referência clínica para futuras intervenções na área da ortopedia

Considerações sobre os Termos de apresentação obrigatória:

Todos os termos foram alterados conforme solicitado em parecer anterior e estão de acordo com os requisitos e orientações do CEP.

Recomendações:

Recomenda-se atenção no cumprimento das exigências da Res. 466/12 e complementares e Guia de Boas Práticas.

Atentar-se ao período e envio de relatórios parciais (semestralmente) e relatório final de estudo ao sistema CEP-CONEP, via plataforma Brasil, para acompanhamento do desenvolvimento do trabalho.

Conclusões ou Pendências e Lista de Inadequações:

As pendências apontadas foram atendidas e/ou justificadas.

Considerações Finais a critério do CEP:

Este parecer foi elaborado baseado nos documentos abaixo relacionados:

Tipo Documento	Arquivo	Postagem	Autor	Situação
Informações Básicas do Projeto	PB_INFORMAÇÕES_BÁSICAS_DO_PROJETO_1458201.pdf	20/02/2020 15:01:35		Aceito
Orçamento	ORCAMENTO_SEGUNDA_REVISAO.pdf	20/02/2020 15:01:22	GABRIEL FERRAZ FERREIRA	Aceito
Projeto Detalhado / Brochura Investigador	PROJETO_PESQUISA_QUINTA_REVISAO.pdf	20/02/2020 15:01:12	GABRIEL FERRAZ FERREIRA	Aceito
Outros	CARTA_RESPOSTA_SEXTA_REVISAO.pdf	20/02/2020 15:00:52	GABRIEL FERRAZ FERREIRA	Aceito
Cronograma	CRONOGRAMA_PRIMEIRA_REVISAO.pdf	20/02/2020 15:00:11	GABRIEL FERRAZ FERREIRA	Aceito
Outros	Termo_Confidencialidade.pdf	20/02/2020 14:59:57	GABRIEL FERRAZ FERREIRA	Aceito
TCLE / Termos de	TCLE_QUINTA_REVISAO.pdf	20/02/2020	GABRIEL FERRAZ	Aceito

Assentimento / Justificativa de Ausência	TCLE_QUINTA_REVISAO.pdf	14:59:08	FERREIRA	Aceito
Outros	Validacao_Gabriel07.pdf	31/01/2020 14:45:09	DANIELA RIMOLDI CUNHA	Aceito
Outros	Validacao_Gabriel06.pdf	24/01/2020 16:40:15	DANIELA RIMOLDI CUNHA	Aceito
Outros	Validacao_Gabriel_05.docx	13/01/2020 15:34:52	DANIELA RIMOLDI CUNHA	Aceito
Outros	CARTA_CEP_29_12_19.pdf	29/12/2019 21:40:11	GABRIEL FERRAZ FERREIRA	Aceito
Outros	Validacao_gabriel04.pdf	20/12/2019 11:09:04	Henrique Guindalini Deliberato	Aceito
Outros	Parecer_IPS.pdf	18/12/2019 17:46:42	GABRIEL FERRAZ FERREIRA	Aceito
Folha de Rosto	FOLHA_DE_ROSTO.pdf	18/12/2019 17:45:02	GABRIEL FERRAZ FERREIRA	Aceito
Outros	Validacao_Gabriel03.pdf	02/12/2019 15:04:56	Henrique Guindalini Deliberato	Aceito
Outros	Validacao_Gabriel02.pdf	11/11/2019 13:58:23	Henrique Guindalini Deliberato	Aceito
Outros	Validacao_Gabriel01.pdf	04/11/2019 16:21:02	Henrique Guindalini Deliberato	Aceito
Declaração de Instituição e Infraestrutura	infraestrutura.pdf	01/11/2019 11:54:02	GABRIEL FERRAZ FERREIRA	Aceito
Outros	APRESENTACAO.pdf	23/10/2019 11:32:00	GABRIEL FERRAZ FERREIRA	Aceito
Declaração de Pesquisadores	Declaracao_do_Pesquisador.pdf	23/10/2019 11:31:31	GABRIEL FERRAZ FERREIRA	Aceito

Situação do Parecer:
Aprovado

Necessita Apreciação da CONEP:
Não

SAO PAULO, 12 de Março de 2020

Assinado por:
PATRICIA ESPINDOLA BRETAS BERBARE (Coordenador(a))
